# Retraction: Curcumin Potentiates Rhabdomyosarcoma Radiosensitivity by Suppressing NF-κB Activity

**DOI:** 10.1371/journal.pone.0222130

**Published:** 2019-08-30

**Authors:** The PLOS ONE Editors

After publication of this article [1], concerns were raised about results reported in the following figures:

- In Figure 1D, data in the following lanes appear similar, when aspect ratio is adjusted for one of the panels:
  - left panel, EV; left panel, IKBα/10Gy; right panel, p100 (flipped horizontally); right panel, IKBα/10Gy
  - left panel, lanes 2, 3, 4, and right panel lanes 2 (flipped horizontally), 1 (flipped horizontally), and 6, respectively
- There appear to be vertical discontinuities between lanes in Figure 1D, 1E.
- In the right panel of Figure 1D, there appear to be discontinuities or abrupt edges within lanes 1 and 2, there are similarities between two areas of the background in lane 1, and an area within the background of lane 1 appears similar to an area within the background of lane 2.
- In Figure 1E, data in the following lanes appear similar:
  - Left panel, lanes 2 and 6
  - Left panel, lanes 3 and 8 (flipped horizontally)
  - Left panel, lanes 4, 5, 7 (flipped horizontally)
  - Right panel, lanes 1 (flipped horizontally), 4, 7
  - Right panel, lanes 2, 3, 6 (flipped horizontally)
- In Figure 2, right panel, lanes 2, 3 appear similar.
- In Figure 3, several lanes appear similar:
  - Figure 3A, left IKBα panel lanes 4, 5
  - Figure 3A, right IKBα panel lanes 1, 5
  - Figure 3A, right IKBα panel lanes 2, 4
  - Figure 3A, left β-actin panel lanes 1, 4, 5 and right β-actin panel lanes 3 (rotated 180) and 5
  - Figure 3A, left β-actin panel lane 2 (aspect ratio adjusted), right β-actin panel lanes 2, 4
  - Figure 3B, left IKBα panel lanes 3, 4
- In Figure 5B, the following lanes appear similar:
  - left p-IKBα panel lanes 1, 4
  - right p-IKBα panel lanes 1, 3
- In Figure S3, there appear to be vertical discontinuities between some lanes, and the following lanes appear similar:
  - MMP-9: Rh30/Combo, Rh41/Control, Rh41/Curcumin, and Rh41/Combo
  - COX-2: Rh30/Control and Rh30/Combo
  - COX-2: Rh41/Curcumin and Rh41/Combo (flipped horizontally)
  - β-actin: Rh41/Control and Rh41/IR
  - β-actin: Rh41/Curcumin and Rh41/Combo
- In Figure S5C, the Control and Radiation panels on the left appear to include a region of overlap.

St. Jude Children’s Research Hospital investigated the irregularities in Figures 1D, 1E, 3, 5B, S3 and S5C and concluded that the data in these figures were fabricated. Following the investigation, the corresponding author requested retraction.

The corresponding author notified *PLOS ONE* that all authors agree to the retraction. The authors apologize to the scientific community for any inconvenience resulting from the publication and retraction of this article.

In light of these issues, the *PLOS ONE* Editors retract the publication.
